# [18F]FDG PET/MRI in rectal cancer

**DOI:** 10.1007/s12149-021-01580-0

**Published:** 2021-01-31

**Authors:** Filippo Crimì, Silvia Valeggia, Luca Baffoni, Roberto Stramare, Carmelo Lacognata, Gaya Spolverato, Laura Albertoni, Alessandro Spimpolo, Laura Evangelista, Pietro Zucchetta, Diego Cecchin, Salvatore Pucciarelli

**Affiliations:** 1 Department of Medicine DIMED, Institute of Radiology, Padova University Hospital, Via Nicolò Giustiniani 2, 35128 Padova, Italy; 2 Radiology Unit, Padova University Hospital, Via Nicolò Giustiniani 2, 35128 Padova, Italy; 3 Department of Surgical, Oncological and Gastroenterological Sciences DiSCOG, 1st Surgical Clinic, Padova University Hospital, Via Nicolò Giustiniani 2, 35128 Padova, Italy; 4 Department of Medicine DIMED, Surgical Pathology and Cytopathology Unit, Padova University Hospital, Via Nicolò Giustiniani 2, 35128 Padova, Italy; 5 Department of Medicine DIMED, Nuclear Medicine Unit, Padova University Hospital, Via Nicolò Giustiniani 2, 35128 Padova, Italy

**Keywords:** Rectal cancer, PET/MRI, [18F]FDG

## Abstract

We conducted a systematic literature review on the use of [18F]FDG PET/MRI for staging/restaging rectal cancer patients with PubMed, Scopus, and Web of Science, based on the PRISMA criteria. Three authors screened all titles and abstracts and examined the full texts of all the identified relevant articles. Studies containing aggregated or duplicated data, review articles, case reports, editorials, and letters were excluded. Ten reports met the inclusion criteria. Four studies examined T staging and one focused on local recurrences after surgery; the reported sensitivity (94–100%), specificity (73–94%), and accuracy (92–100%) varied only slightly from one study to another. The sensitivity, specificity, and accuracy of [18F]FDG PET/MRI for *N* staging were 90–93%, 92–94%, and 42–92%. [18F]FDG PET/MRI detected malignant nodes better than MRI, resulting in treatment change. For *M* staging, [18F]FDG PET/MRI outperformed [18F]FDG PET/CT and CT in detecting liver metastases, whereas it performed worse for lung metastases. The results of this review suggest that [18F]FDG PET/MRI should be used for rectal cancer restaging after chemoradiotherapy and to select patients for rectum-sparing approaches thanks to its accuracy in *T* and *N* staging. For *M* staging, it should be associated at least with a chest CT scan to rule out lung metastases.

## Introduction

Colorectal cancer is the most common gastro-intestinal cancer and the third leading cause of cancer-related death worldwide. Rectal cancer accounts for about one in three cases of colorectal tumor [1] and is clinically suspected mainly from evidence of bloody stools, symptoms of obstruction, anemia or polypoid lesions on colonoscopy [2].

Initial staging should provide information regarding: rectal wall invasion and involvement of adjacent organs or important anatomical structures (*T* stage), loco-regional nodal involvement (*N* stage), and distant metastases (*M* stage) [3]. Patients with locally advanced rectal cancer (*TNM* stages II or III) are at high risk of local recurrence and should undergo preoperative chemo-radiotherapy (pCRT) [4]. Disease staging is therefore mandatory to tailor the most appropriate treatment strategy to each patient. The standard of care for locally-advanced rectal cancer currently includes pCRT followed by radical surgery (total mesorectal excision) with curative intent, and adjuvant chemotherapy [5, 6].

Accurate staging also plays an irreplaceable part in relation to the recent introduction of rectum-sparing approaches (transanal local excisions or watch-and-wait protocols) as alternatives to conventional surgery for patients showing a major or complete clinical response to pCRT on restaging [7, 8]. Current guidelines recommend MRI for local staging, and contrast-enhanced thoraco abdominal CT for detecting distant metastases [9].

[18F]FDG PET/CT is reportedly a good predictor of complete response after pCRT [10], performing better than conventional CT in identifying distant metastases [11]. No comparable evidence of its value in nodal staging has been reported so far [12].

Combined [18F]FDG PET/MRI has recently been proposed as an effective imaging modality for rectal cancer patients, capable of generating high-resolution anatomical and functional data. This combined imaging modality can also spare patients the radiation exposure [13] associated with the CT component of PET/CT. In fact, a mean dose reduction in the range of 7.40–9.16 mSv has been reported for PET/MRI compared with PET/CT [14]. PET/MRI also achieves a high soft-tissue contrast that is useful for examining solid organs, such as the liver [15], and it can be implemented with specific MRI sequences, such as diffusion-weighted imaging (DWI), if necessary [16].

The combination of the above-mentioned complementary advantages of PET and MRI might theoretically improve our diagnostic accuracy and treatment decision-making for rectal cancer [15]. A wide consensus on the clinical usefulness of [18F]FDG PET/MRI in the staging and restaging of rectal cancer patients has yet to be reached, however. Hence our present study aimed to conduct a systematic literature review and to analyze publications on the use of [18F]FDG PET/MRI for staging or restaging rectal cancer patients.

## Methods

### Search strategy

PubMed, Web of Science, and Scopus were used for our literature search, from inception through to December 2019, based on the PRISMA criteria [17]. The following terms and their variants were used in the title, abstract and keyword fields, and MeSH fields where available, adapting the search syntax where necessary: “PET/MRI AND rectal AND cancer; PET-MRI AND rectal AND cancer; PET AND MRI AND rectal AND cancer; PET/MRI AND rectal AND tumor; PET-MRI AND rectal AND tumor; PET AND MRI AND rectal AND tumor; PET/MRI AND rectal AND tumour; PET-MRI AND rectal AND tumour; PET AND MRI AND rectal AND tumour”.

The reference lists of the articles included in this review, and narrative reviews published in the last 10 years were also searched manually for articles not identified by the initial literature search.

Our selection criteria excluded studies not written in English, studies containing aggregated data or data duplicated from previously published work, and review articles, case reports, editorials, and letters. No restrictions were placed on study design or population.

### Systematic review protocol and data extraction

Three authors (S.V., F.C., and L.B.) independently screened all titles and abstracts generated by the first search, then examined the full texts of all relevant articles identified according to the inclusion criteria.

Any disagreement regarding the article suitability was settled by a discussion between the assessors or, failing this, by referral to a fourth senior author (D.C.).

**Fig. 1:**
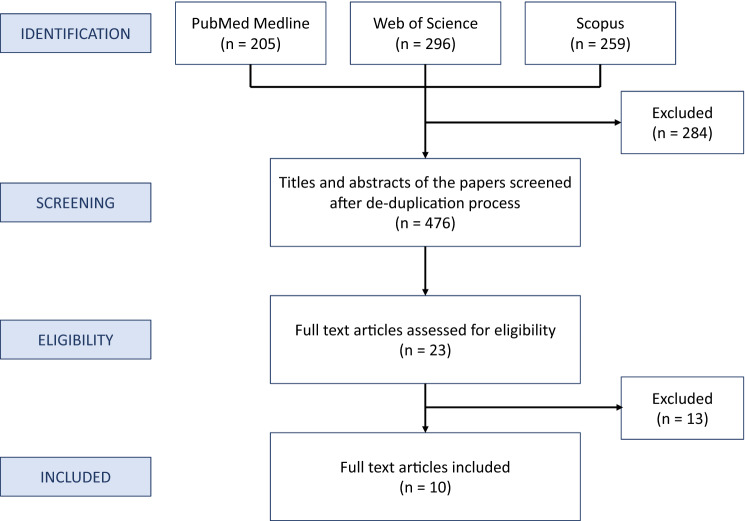
The literature review flowchart

Among 476 studies obtained from the literature search, 10 reports published between June 2015 and December 2019 met the inclusion criteria (Fig. 1). Data from these studies were extracted using a standardized pro forma in Microsoft Excel (Redmond, WA, USA). The information extracted from each study included: author, year of publication, study design, number of cases, and other characteristics shown in Table 1.

**Table 1:** Studies included in the review

Author, ref	Year of pub	Study design	*N* of pts	Setting of disease (no. of pts)	Comparison with contrast-enhanced MRI/CT	Comparison with PET/CT	PET interpretation	Gold standard (no. of pts)
Bailey et al. [18]	2018	R	22 rectum	*s* (22)	No	No	Visual analysis	None
Brendle et al. [19]	2016	R	9 colon 4 sigmoid 2 rectum	*s* (1) *r* (14)	MRI	Yes	Visual analysis	Histopathology (10) Imaging (5)
Crimì et al. [20]	2019	P	36 rectum	*s* (36)	MRI/CT	No	Visual analysis	Histopathology Imaging
Jeong et al. [16]	2016	P	9 rectum	*s* (9)	No	Yes	Visual and semiquantitative analysis	None
Kang et al. [21]	2016	R	28 colon 23 rectum	*s* (32) *r* (19)	CT	No	Visual analysis	Histopathology (12) Imaging (39)
Lee et al. [22]	2015	P	30 colon 29 rectum	*s* (20) *r* (39)	MRI	No	Visual analysis	Histopathology Imaging
Paspulati et al. [23]	2015	P	12 colon-rectum	*s* (2) *r* (10)	No	Yes	Visual and semiquantitative analysis	Histopathology (8) Imaging (2) Clinical (10)
Plodeck et al. [24]	2019	R	44 rectum	*r* (44)	No	No	Visual analysis	Histopathology (27) Imaging (17)
Rutegård et al. [25]	2019	P	24 rectum	*s* (24)	No	Yes	Visual analysis	Clinical (24)
Yoon et al. [26]	2019	P	71 rectum	*s* (71)	MRI/CT	No	Visual analysis	Imaging (71)

*R* retrospective, *P* prospective, *s* staging before or after preoperative chemoradiotherapy, *r* restaging after surgery or suspect of recurrent disease, *NA* not available, *NAC* neoadjuvant chemotherapy (or chemo radiation therapy), *CT* computed tomography, *MRI* magnetic resonance imaging, *PET/CT* positron emission tomography/computed tomography

## Results

Bailey et al. [18] investigated whether extended PET acquisition times in the pelvis using [18F]FDG PET/MRI could increase the detection rate of potentially metastatic lymph nodes in rectal cancer patients. Twenty-two patients (drawn from among 29 studies) with biopsy-proven rectal adenocarcinoma underwent whole-body simultaneous [18F]FDG PET/MRI study with a mean FDG dose of 300.8 ± 59.2 MBq, and image acquisition 72.8 ± 22.3 min after the injection. The protocol included a 3-min and a 15-min PET/MRI acquisition of the pelvis. The 15-min PET acquisition of the pelvis enabled the identification of roughly 40% more FDG-avid lymph nodes than the classic 3-min acquisition. It was recommended as a useful addition to the dedicated pelvis MRI protocol for rectal cancer staging. There was also less inter-reader variability on PET/MRI than on MRI alone.

Brendle et al. [19] compared [18F]FDG PET/CT with a consecutively acquired [18F]FDG PET/MRI, including DWI, in 15 colorectal cancer patients, conducting a separate analysis for mucinous tumors. PET/MRI/DWI was comparable with PET/CT in terms of overall accuracy, more accurate for detecting liver lesions (74% vs. 56%), and equally accurate for peritoneal and lymph node metastases.

Crimì et al. [20] examined the restaging of 36 patients with locally advanced rectal cancer after pCRT, highlighting a slightly higher accuracy in T (92% vs. 89%) and N staging (92% vs. 86%) for whole-body [18F]FDG PET/MRI than for MRI alone. PET/MRI findings also prompted changes to the treatment plan in 11% of cases when hypermetabolic tumor residuals were detected among the areas of fibrosis.

Jeong et al. [16] measured the mean apparent diffusion coefficient (ADC) and the max/peak/mean standardized uptake values (SUV) of rectal tumors in a sample of nine patients with rectal adenocarcinomas undergoing both [18F]FDG PET/CT and PET/MRI sequentially. Even though mean SUVmax, SUVpeak and SUVmean values of the primary lesions obtained by hybrid PET/MRI were significantly lower than SUVs determined by PET/CT, the quantitative evaluation of PET images revealed high correlation between maximum, peak and mean SUVs obtained using the two modalities (SUVmax, *ρ* = 0.82, *p* = 0.007; SUVpeak, *ρ* = 0.93, *p* < 0.001; SUVmean, *ρ* = 0.77, *p* = 0.016). A significant inverse correlation between the ADC and the max/peak/mean SUV emerged in hybrid PET/MRI, suggesting an association between the tumor’s metabolic activity and water diffusivity, and a complementary role of these two parameters in rectal cancer staging.

Kang et al. [21] compared the diagnostic value of [18F]FDG PET/MRI with contrast-enhanced CT in 51 patients with colorectal cancer. The PET/MRI protocol included additional MRI images of any organs suspected of harboring secondary lesions.

PET/MRI provided more information than CT in 27.5% of patients, proving particularly useful for characterizing lesions indeterminate on CT in the liver (*n* = 7), at the surgical site (*n* = 4), and in the lungs (*n* = 1), and for identifying additional malignant lesions in the liver not visible on CT (in 3.9% of patients). PET/MRI consequently led to the treatment strategy being adjusted for 21.6% of patients. Compared with CT, PET/MRI was less able to reveal small metastatic lesions in the lung; it detected 52.9% of the pulmonary metastatic nodules visible on CT.

Lee et al. [22] tested the diagnostic accuracy of a protocol consisting of a whole-body FDG PET/Dixon-VIBE MRI, followed by dedicated MRI (enhanced and unenhanced) of the liver in cases of suspected secondary lesions. Fifty-nine patients with colorectal cancer were considered. For primary colorectal cancers (*n* = 14), the protocol proved highly sensitive (100%) in detecting primary lesions, highly accurate in *T* staging (93%), and very sensitive in *N* (93%) and *M* (100%) staging. In 38 patients with suspected metastatic liver lesions, its sensitivity varied from 94.4% (before treatment) to 75% (after neoadjuvant treatment). In short, the study demonstrated that PET/MRI performed well in staging/restaging colorectal cancer and hepatic metastases of chemo-naïve patients.

Paspulati et al. [23] compared the diagnostic accuracy of [18F]FDG PET/MRI and [18F]FDG PET/CT in a sample of 12 patients. PET/MRI showed a better diagnostic accuracy in *T* staging and an at least comparable diagnostic value in *N* and *M* staging. On a per-patient basis, the true positive rate was 71% for PET/CT and 86% for PET/MRI, while the true negative rate was 100% for both imaging modalities.

According to Plodeck et al. [24], [18F]FDG PET/MRI seems to be good at revealing pelvic recurrences of rectal cancer, and has an important role in the diagnosis and management of this disease. It demonstrated a high sensitivity (94%), specificity (94%), and accuracy (94%) in a pool of 44 patients (and 47 examinations). Two false negative cases came to light, both in patients whose PET/MRI was performed after neoadjuvant chemotherapy; and in one of these patients the histological subtype was mucinous adenocarcinoma. The PET/MRI results prompted adjustments to the treatment strategy in eight patients, and previous imaging studies on five of these patients had revealed no or uncertain malignant lesions.

Rutegard et al. [25] investigated the role of adding hybrid imaging ([18F]FDG PET/MRI and [18F]FDG PET/CT) in the staging and restaging of rectal cancer in 24 patients. In one case, PET alone detected a liver metastasis, thus upstaging the patient to *M*1 and prompting a change of therapeutic approach that led to a hepatic metastasectomy.

Yoon et al. [26] studied the diagnostic yield of FDG PET/MRI for the purpose of *M* staging in a pool of 71 patients with newly diagnosed advanced mid-to-low rectal cancer. The [18F]FDG PET/MRI examination included additional dedicated MRI sequences of the liver (without and with gadoxetic acid) and the rectum. It was compared with a “standard of care” (SOC) protocol that included contrast-enhanced chest and abdominopelvic CT and rectal MRI. Overall specificity was much higher for PET/MRI (98%) than for SOC (73%), and it increased to 100% (vs. 88%) in patients with inconclusive findings regarding *M* stage using SOC. PET/MRI proved very helpful in excluding metastatic disease thanks to its high specificity, without raising the risk of missing secondary lesions (overall sensitivity was 94% for both PET/MRI and SOC). PET/MRI also enabled a better characterization of most of the incidental findings emerging from SOC, including additional neoplasms.

### Results by *T*, *N*, and *M* stages

#### *T* staging

Among the reviewed studies, four [20–23] examined *T* staging with [18F]FDG PET/MRI, and one focused on local recurrences after surgery [24], comparing the findings with PET/CT. All but two studies [21, 24] were prospective. Two authors looked at restaging and focused mainly on patients who had undergone pCRT [20, 22]. One study examined pelvic recurrences [24], and two others considered both staging and restaging after treatment [21, 23]. The reported sensitivity (94–100%), specificity (73–94%), and accuracy (92–100%) of [18F]FDG PET/MRI varied only slightly from one study to another (Table 2). The highest accuracy (100%) of *T* staging with [18F]FDG PET/MRI was found in a pool of 12 patients with no previous chemo-radiotherapy by Kang et al. [21]. In 44 patients with local recurrences of rectal cancer, Plodeck et al. [24] found much the same rates of sensitivity (94%), specificity (94%) and accuracy (94%). False negative results were mainly due to recent effects of pCRT, or specific histological subtypes (such as mucinous tumors).

**Table 2:** Diagnostic accuracy for *T*, *N*, and *M* staging reported in the different studies

Author, ref	No. of pts	*TNM* staging	Sensitivity	Specificity	Accuracy
Bailey et al. [18]	22	–	–	–	–
Brendle et al. [19]	15	Non-mucinous s/rTNM	68%	88%	76%
		Mucinous s/rTNM	38%	85%	52%
Crimì et al. [20]	36	T (after pCRT)	100%	73%	92%
		N (after pCRT)	90%	92%	92%
Jeong et al. [16]	9	–	–	–	–
Kang et al. [21]	51	sT	–	–	100%
		sN	–	–	42%
Lee et al. [22]	59	sT	–	–	93%
		sN	93%	–	–
		sM (liver)	100%	–	–
		rM (liver)	75%	–	–
Paspulati et al. [23]	12	rN and rM	86%	100%	95%
Plodeck et al. [24]	44	rTNM	94%	94%	94%
Rutegård et al. [25]	24	–	–	–	–
Yoon et al. [26]	71	sM	94%	98%	–

*T* tumor, *N* nodal, *M* metastases, *s* staging before or after preoperative chemoradiotherapy, *r* restaging after surgery or suspect of recurrent disease, *pCRT* preoperative chemoradiation therapy

In a restaging setting, two authors [20, 22] found PET/MRI slightly superior to MRI for *T* staging purposes (with a sensitivity of 92.9% and 92.3%, and an accuracy of 92% and 89%, respectively).

**Fig. 2:**
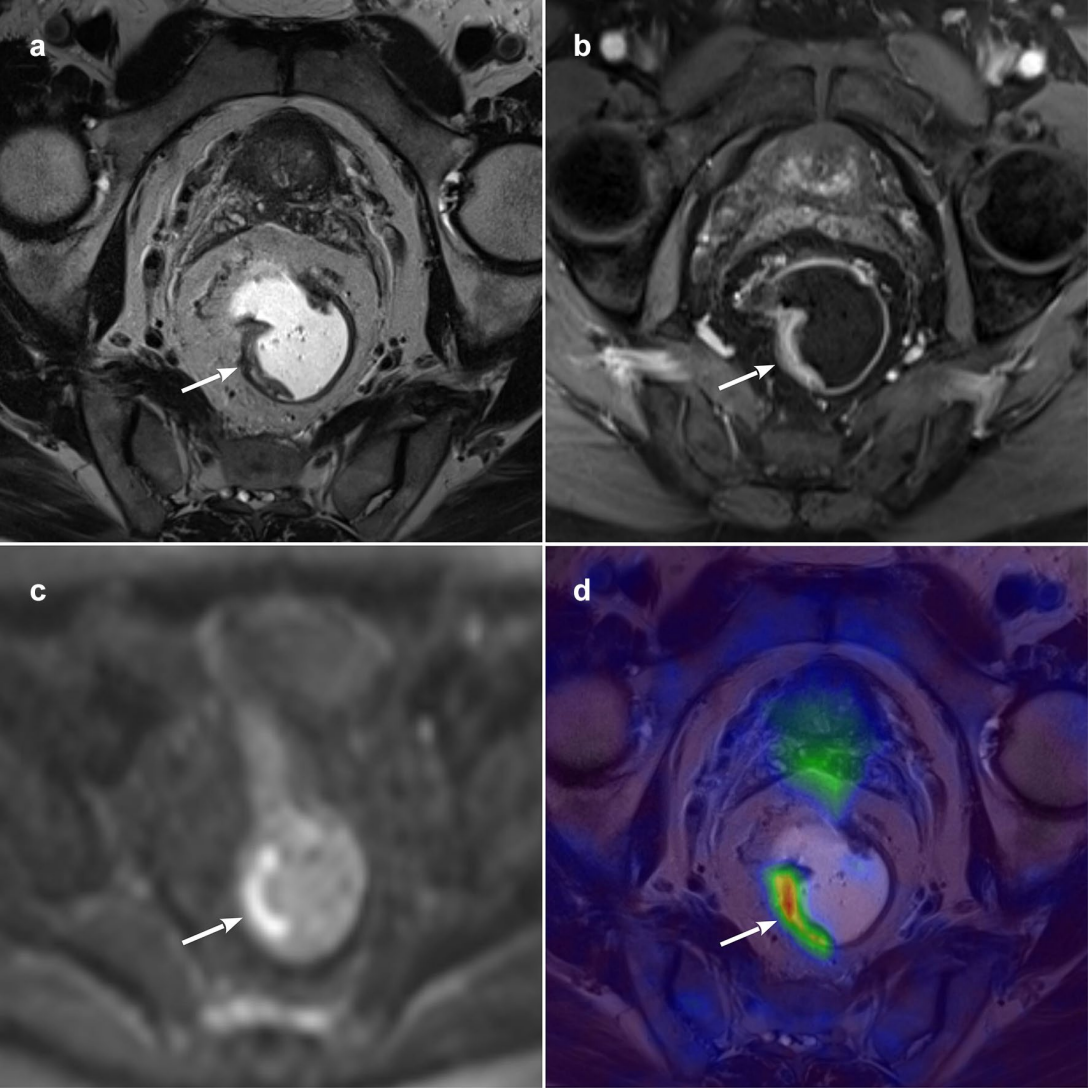
PET/MR images of a histologically-proven T2 rectal cancer after pCRT. **a** *T*2-weighted paraxial image showing an irregular thickening of the right rectal wall (arrow), with no signs of extramural invasion; **b** paraxial contrast-enhanced volumetric interpolated breath-hold examination (VIBE) with irregular enhancement of the same lesion (arrow); **c** axial b1000 diffusion-weighted imaging (DWI) showing signal restriction of the mass (arrow); **d** paraxial PET/MR fused image with hypermetabolism of the rectal tumour (arrow)

In the study by Paspulati et al. [23], PET/MRI proved more efficient than PET/CT. For example, it enabled detailed *T* staging and revealed mesorectal fascia involvement in a patient with a *T*3 lesion not seen on [18F]FDG PET/CT. It also improved the identification of hypermetabolic tumor remnants among the areas of fibrosis after chemo radiotherapy, eventually prompting changes in therapeutic strategy [20] (Fig. 2).

#### *N* staging

Four studies analyzed *N* staging with [18F]FDG PET/MRI [20–23], three of them prospective [20, 22, 23], and one retrospective [21]. One study examined *N* staging performance before pCRT in a subgroup of patients [21], two after chemo radiation [20, 22], and one considered a group of patients both before and after pCRT [23]. The sensitivity of [18F]FDG PET/MRI in detecting nodal involvement ranged from 90 to 93% [20, 22], and its specificity was very similar in all four studies (92–94%), while its accuracy varied from 42 to 92% [20, 21] (Table 2). Kang et al. [21] reported the lowest accuracy, probably because they compared the results of imaging with histopathological findings on a stage-based approach, with *N* staging according to the American Joint Committee on Cancer (AJCC) classification (*N*0, *N*1, and *N*2), instead of a per-patient approach (loco-regional node-positive vs. node-negative).

**Fig. 3:**
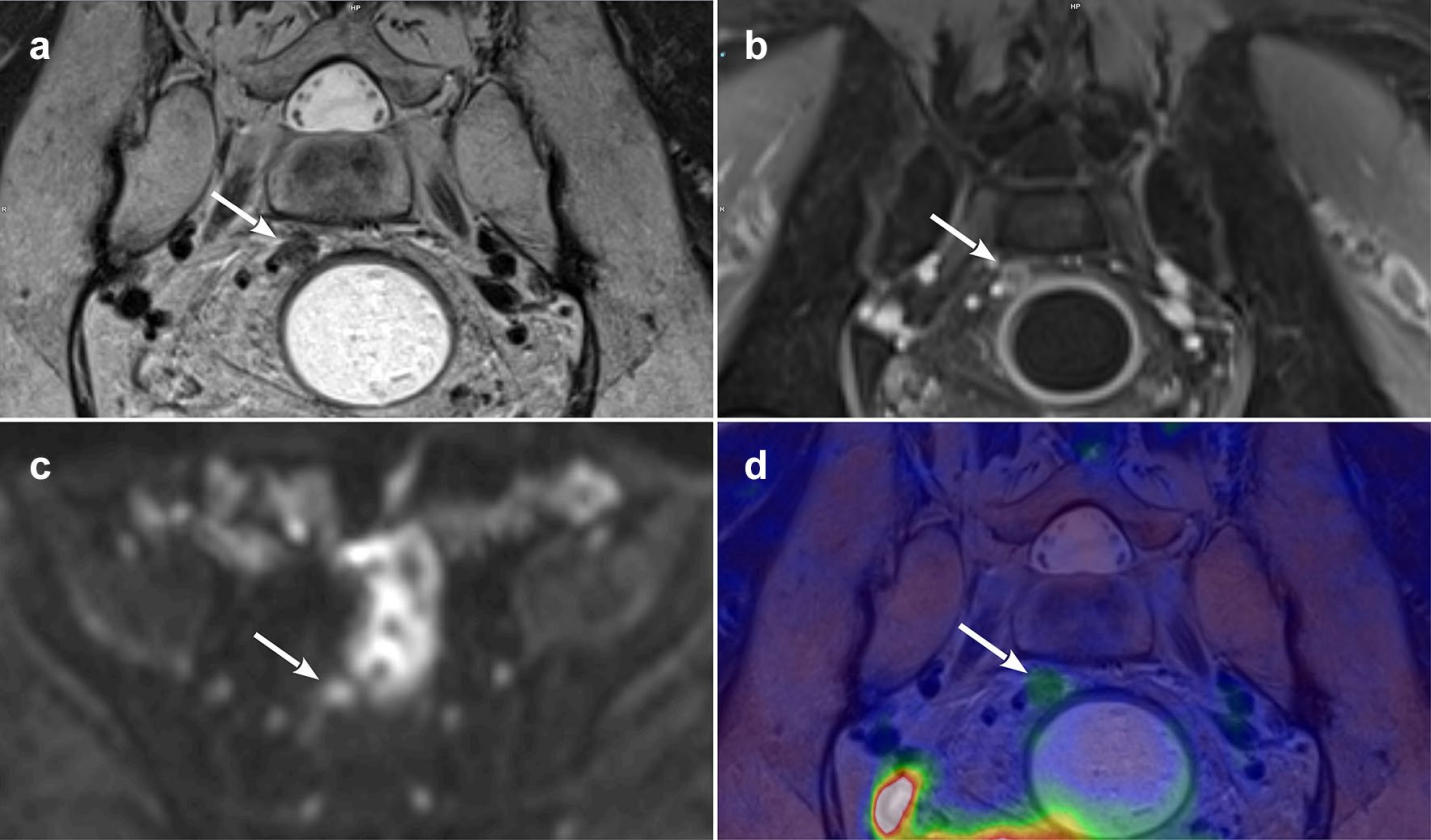
PET/MR images of a malignant mesorectal lymph node after pCRT. **a** *T*2-weighted paracoronal image showing rounded lymph node with short axis of 6 mm, irregular margins and internal signal heterogeneity (arrow); **b** paracoronal contrast-enhanced VIBE showing the same lymph node (arrow) with internal signs of necrosis; **c** axial b1000 diffusion-weighted imaging (DWI) identifying the lymph node (arrow); **d** paracoronal PET/MR fused image with slight hypermetabolism of the small lymph node (arrow)

When two of the studies compared PET/MRI and MRI for *N* staging purposes one of them [22] found a comparable sensitivity of the two techniques (92.9%), while the other [20] reported a superiority of PET/MRI over MRI (with an accuracy of 92% vs. 86%). Paspulati et al. [23] found the accuracy of PET/CT and PET/MRI similar in the detection of regional lymph node metastases. Combined [18F]FDG PET/MRI has been shown to detect hypermetabolic lymph nodes better than MRI alone, leading to treatment changes as a result [20] (Fig. 3).

#### *M* staging

**Fig. 4:**
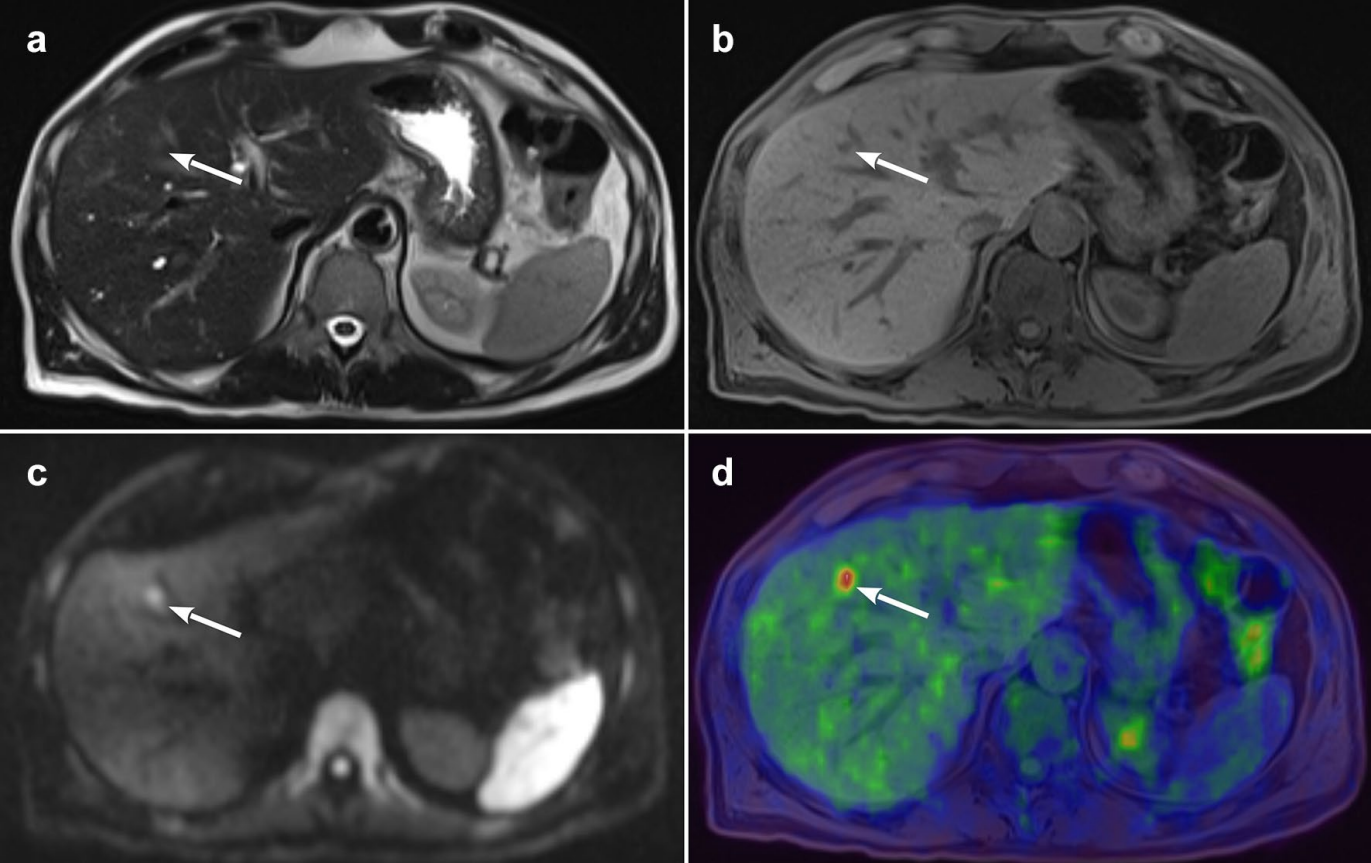
PET/MR images of a small liver metastasis. **a** *T*2-weighted axial image showing small area of slight hyperintensity in S8 (arrow); **b** axial VIBE with a small hypointense lesion of 7 mm in S8 (arrow); **c** axial b1000 diffusion-weighted imaging (DWI) confirming the signal restriction of the mass (arrow); **d** axial PET/MR fused image with hypermetabolism of the liver lesion (arrow)

Five studies examined the accuracy of [18F]FDG PET/MRI for the purpose of *M* staging [19, 21, 22, 25, 26], three of them prospective [22, 25, 26] and two retrospective [19, 21] (Fig. 4).

With a sensitivity of 96% in detecting metastases, [18F]FDG PET/MRI proved more useful before CRT than afterwards (post-CRT its sensitivity was 75%), according to Lee et al. [22]. Another study by Brendle et al. [19] also found a lower overall sensitivity in detecting secondary lesions in the liver (71%) or lymph nodes (47%) when they considered patients enrolled for both staging and restaging after pCRT, and those with mucinous subtypes of cancer. Brendle et al. [19] also reported on the specificity (80%) and accuracy (74%) of [18F]FDG PET/MRI in detecting liver metastases.

Two other studies [21, 26] showed that the added value of [18F]FDG PET/MRI in detecting distant metastases that other imaging techniques overlooked or judged indeterminate helped to orient treatment decisions and optimize its timing. Yoon et al. [26] found that [18F]FDG PET/MRI imaging improved *M* staging in 21/22 patients revealing indeterminate lesions on contrast-enhanced CT. The high overall specificity (98%) of [18F]FDG PET/MRI for *M* staging purposes was especially helpful in ruling out secondary lesions, with consequences for clinical management. On the other hand, its sensitivity in detecting metastases (94%) was similar to that of SOC (CT + MRI) mainly due to its poor rate of detection for small lung nodules [26].

Kang et al. [21] demonstrated that PET/MRI provided additional information vis-à-vis the findings of other imaging modalities (such as CT) in a fair number of patients (27.5%). Compared with PET/CT, PET/MRI was also able to identify three more metastatic lesions involving the rectal and perirectal region in one study on 12 patients [23], and one additional metastatic lesion in a sample of 24 patients in another report [25].

## Discussion

Pooling the information from the above-mentioned studies, we summarized that [18F]FDG PET/MRI is better than [18F]FDG PET/CT or MRI, especially in a restaging setting. For *T* and *N* staging purposes, [18F]FDG PET/MRI proved slightly better than MRI, but comparable with [18F]FDG PET/CT. For *M* staging, on the other hand, the strength of [18F]FDG PET/MRI—by comparison with [18F]FDG PET/CT and CT imaging—lies in its performance in detecting liver metastases, whereas it performed less well than other techniques for lung metastases. Another strength of [18F]FDG PET/MRI—again compared with [18F]FDG PET/CT—concerns the lower exposure to ionizing radiation [14].

The initial studies reviewed here show that [18F]FDG PET/MRI performs better in TNM staging than [18F]FDG PET/CT or MRI/SOC alone. In fact, the anatomic information provided by the low-dose, non-contrast CT component of the PET/CT examination is often insufficient to determine the extent of local tumor invasion or to characterize incidental lesions; instead, the superior soft-tissue contrast resolution, the multiplanar imaging acquisition capability, and the additional value of functional sequences such as diffusion-weighted imaging, allow better visualization of soft tissue and musculoskeletal structures, which in turn enhances the diagnostic efficiency of PET/MRI over PET/CT or CT alone. Its greater diagnostic accuracy suggests a pivotal role for [18F]FDG PET/MRI in orienting the approach to therapy for rectal cancer. In fact, PET/MRI findings reportedly prompted changes to the patient treatment strategy in three of the examined studies [20, 21, 26].

[18F]FDG PET/CT was able to predict a histopathologically complete response after pCRT for rectal cancer with a sensitivity and specificity of 71% and 76%, respectively [10]. For restaging purposes, [18F]FDG PET/MRI combined the already-reported good accuracy of PET images with the anatomical detail of MRI, enabling a better *T* staging [20–23]. It has shown that the tumor response to pCRT often consists of partial or complete replacement of viable tumor tissue with fibrosis; consequently, tumor under-staging in this setting might be due to the inability of PET/CT to detect small clusters of residual disease or failure of morphological sequences (such as conventional *T*2-weighted MRI) and functional ones (such as diffusion-weighted imaging) to identify small remnant tumor deposits in the initial tumor site. By combining morphological, functional and metabolic data, PET/MRI appears to be able to accurately re-stage patients after pCRT [20]. The reported limitations of [18F]FDG PET/MRI concern the difficulty of detecting mucinous lesions (probably due to their low FDG avidity [19]), and residual lesions after pCRT (due to the confounding low-grade uptake of post-irradiation fibrous tissue).

MRI alone has some limitations when it comes to *N* staging in case of rectal cancer and in discriminating between benign and malignant loco-regional nodes [27]. As for *N* staging, a meta-analysis by Lu et al. [12] had not found sufficient evidence to support the routine clinical use of [18F]FDG PET/CT for *N* staging in colorectal cancer, since it achieved a sensitivity of 42.9% and a specificity of 87.9%. In the group of articles analyzed here, the accuracy of [18F]FDG PET/MRI for *N* staging purpose ranged between 42 and 92% [20–23]; in other words, its accuracy was basically the same or only slightly superior to that of MRI, and comparable with [18F]FDG PET/CT.

The analyzed studies confirm that the soft-tissue contrast and resolution of PET/MRI can be very useful when applied to abdominal solid organs, such as the liver [13]. PET/MRI showed an advantage over PET/CT or contrast-enhanced CT in characterizing liver lesions that would have otherwise been too small to characterize or remained indeterminate. This is thanks to the opportunity to obtain diffusion-weighted imaging sequences and use hepatocyte-specific contrast media, which can detect lesions in the hepatobiliary phase, providing an excellent contrast between liver and lesion [16, 19, 21, 22, 25, 26].

PET/MRI can have an important role in identifying the distant metastases of rectal cancer, despite the known intrinsic weakness of MRI in identifying the metastatic lung lesions (even after the introduction of new dedicated MRI sequences) [28, 29]. The limited ability of MRI (compared with CT) to identify small lung nodules is reflected in the co-acquired PET findings. There are three main reasons for this: (1) motion artifacts due to cardiac or respiratory movements; (2) the relatively low proton density of lung parenchyma, with a consequently low signal-to-noise ratio; and (3) susceptibility to artifacts related mainly to the multiple interfaces between air and lung tissues. New, specific sequences have recently been developed to address this issue. The use of ultra-short echo times (UTE) sequences could facilitate the visualization of lung parenchyma, achieving a greater sensitivity [28] in the detection of lesions [29]. An acceptable diagnostic accuracy has yet to be reached, however.

Moreover, it is worth noting that FDG uptake, as displayed by SUV measurements, is only partially comparable between PET/MRI and PET/CT [30], and this needs to be borne in mind when comparing PET/CT vs. PET/MRI studies.

Finally, there were differences among the examined studies in the time interval between FDG injection and PET acquisition (60–120 min), in the duration of each PET bed for the whole-body scan (2–8 min) and in the duration of the pelvis dedicated PET bed (15–42 min). These factors might have influenced the FDG uptake of malignant lesions through the different studies and, therefore, the diagnostic accuracy of the techniques.

The main limitations of PET/MRI are the long acquisition times (33–85 min) and the expensive cost of the examination.

The conclusions that can be drawn from our review unfortunately have several limitations. First of all, four of the studies included in the review grouped rectal and colon cancers together, though the approach to their staging and treatment may differ. Second, some studies considered a mixed population of patients examined before and after pCRT, or with different histopathological subtypes (mucinous and non-mucinous tumors). Finally, the size of the samples analyzed in the studies was generally small.

## Conclusion

Although the role of [18F]FDG PET/MRI in rectal cancer has yet to be established, the evidence pooled in this review suggests the following indications for [18F]FDG PET/MRI in the management of rectal cancer patients.

1. It should be used mainly for rectal cancer restaging after pCRT, or for identifying recurrences.
2. It could be a precise tool for choosing which patients to address to rectum-sparing approaches instead of classic surgery because of its accuracy in *T* staging and *N* staging, compared with PET/CT or MRI.
3. When used for M staging, it should be associated with at least a chest CT scan to rule out any lung metastases.

The MRI examination protocol that could be suggested to maximize the diagnostic accuracy of PET/MRI while keeping an acceptable acquisition time should include: (1) whole body *T*2-weighted and *T*1-weghted sequences plus a sequence for lung parenchyma evaluation; (2) a pelvic protocol with *T*2-weighted sequences in three planes, DWI and contrast enhanced sequences; (3) a dedicated protocol for the upper-abdomen preferably after injection of hepato-specific contrast agent.

The heterogeneity of the included studies did not allow us to perform a meta-analysis of the performance of [18F]FDG PET/MRI in rectal cancer. However, the present review offers a complete overview of the literature about the topic.

## Data Availability

The datasets generated during and/or analysed during the current study are available from the corresponding author on reasonable request.
